# Diagnostic Performance of Toluidine Blue Stain for Direct Wet Mount Detection of *Cryptosporidium* Oocysts: Qualitative and Quantitative Comparison to the Modified Ziehl–Neelsen Stain

**DOI:** 10.3390/diagnostics13152557

**Published:** 2023-08-01

**Authors:** Shahira Abdelaziz Ali Ahmed, Annalisa Quattrocchi, Sherine M. Elzagawy, Panagiotis Karanis, Samer Eid Mohamed Gad

**Affiliations:** 1 Department of Parasitology, Faculty of Medicine, Suez Canal University, Ismailia 41522, Egypt; sherriola@hotmail.com (S.M.E.); samargad@med.suez.edu.eg (S.E.M.G.); 2 Department of Primary Care and Population Health, University of Nicosia Medical School, 21 Ilia Papakyriakou, 2414 Engomi, Nicosia CY-1700, Cyprus; quattrocchi.a@unic.ac.cy; 3 College of Health and Rehabilitation Sciences, Princess Nourah Bint Abdulrahman University, Riyadh 11671, Saudi Arabia; 4 Medical Faculty and University Hospital, University of Cologne, 50923 Cologne, Germany; 5 Department of Basic and Clinical Sciences, University of Nicosia Medical School, 21 Ilia Papakyriakou, 2414 Engomi, Nicosia CY-1700, Cyprus

**Keywords:** *Cryptosporidium*, staining, wet mount technique, toluidine blue, modified Ziehl–Neelsen

## Abstract

(1) Background: The wet mount staining technique is a simple, economical, and rapid method for detecting parasitic stages. The objective of the current study was to evaluate wet mount diagnostic stains for *Cryptosporidium* infection in human faecal samples and to compare the sensitivity and qualitative performance of toluidine blue (TolB) and modified Ziehl–Neelsen (mZN) stain. (2) Methods: The collection, purification, and molecular amplification of *Cryptosporidium* oocysts were performed. TolB, malachite green, trypan blue, and crystal violet were evaluated qualitatively to diagnose *Cryptosporidium* oocysts. The outperforming stain was compared to mZN using a quantitative evaluation and qualitative scoring system. (3) Results: The oocysts of *Cryptosporidium parvum* were effectively purified and utilized for spiking. TolB was the most effective diagnostic stain among wet mount stains for detecting *Cryptosporidium* oocysts. TolB outperformed mZN in terms of sensitivity, with 100% versus 79% at a concentration of 10^4^ and 49% versus 23% at a concentration of 10^2^. TolB had the most favourable qualitative stain characteristics against mZN; however, sample freshness and being a temporary stain were crucial considerations. (4) Conclusions: This study emphasizes that TolB, as a routine wet mount technique for screening *Cryptosporidium* infection, will provide a more sensitive and faster method than mZN staining.

## 1. Introduction

Standard ova and parasite examination (O&P) failed to discover *Cryptosporidium* sp., a protozoan intestinal parasite [1]. *Cryptosporidium* sp. is the third most pathogenic protist affecting the African population, with the highest estimated prevalence ranging from 21 to 50% [2]. *Cryptosporidium* was the most often reported waterborne protozoa in African water, accounting for fifty per cent of reports [3]. Statistics on the prevalence of *Cryptosporidium* sp. throughout Asia vary widely, from 0.3% to 70%, depending on the population and country of the study [4]. In organ transplant recipients, the prevalence of *Cryptosporidium* infection reached 15%, with a higher incidence in adults with diarrhoea and studies conducted in developing countries [5]. As many as 905 water and 25 foodborne outbreaks were traced back to *Cryptosporidium* contamination [6,7,8,9].

It is known that *Cryptosporidium* sp. can be disseminated through ingesting contaminated food or drink, through the spread of zoonotic diseases, through intimate human contact, and the less well-known route of airborne transmission [3,9,10]. The *Cryptosporidium* parasite has at least 47 recognized species [11,12] and over 120 confirmed genotypes [13]. Nearly 20 species have been identified in humans, with *Cryptosporidium hominis* (*C. hominis*) and *Cryptosporidium parvum* (*C. parvum*) being the most significant [14]. 

Despite the availability of several diagnostic procedures for detecting *Cryptosporidium* in faeces, slaughterhouses, food, and water [7,10,15,16,17], diagnosis of *Cryptosporidium* sp. is still absent in many countries, for example, in Egyptian [1] and Cyprus laboratories [18], although it is present in any country. Numerous characteristics, including the tiny, microscopic size, staining absorbance, and shape of *Cryptosporidium* sp. oocysts, may contribute to the difficulty in diagnosis, as reviewed by Ahmed and Karanis (2018) [15].

In developing countries, where testing for *Cryptosporidium* sp. is performed depending on modified Ziehl–Neelsen (mZN), the infection is often misdiagnosed or undetected, leading to severe epidemiological problems and a heavy burden [1,2,10]. The mZN stain has been reported as the gold standard for detecting *Cryptosporidium* sp. oocysts, being a proper permanent stain [19,20,21]. The procedure is straightforward, moderately time-consuming (approximately 30 to 45 min), but requires extensive training and experience to interpret the findings [15,21,22,23]. Distinguishing *Cryptosporidium* oocysts from other faecal elements, such as moulds and yeast, is a typical disadvantage in the mZN technique [22,23]. Oocyst staining is also variable. In particular, infections that are resolving can contain colourless “ghosts” oocysts [24]. Compared to nested PCR, the sensitivity, specificity, and positive and negative predictive values of mZN to detect *Cryptosporidium* oocysts in children’s faecal specimens were reported to be 94%, 100%, 100%, and 98%, respectively [20].

Numerous other stains detect *Cryptosporidium* sp. in stool samples with variable advantages and disadvantages [15]. The negative staining technique of Heine is another simple and efficient non-permanent stain of *Cryptosporidium* oocysts [21,25]. Nigrosin staining, light-green staining, and malachite green were also used as negative stain that stained background yeasts and bacteria but not oocysts [26]. The Heine negative stain is modified using other reagents, such as malachite green, methylene blue, and crystal violet, instead of carbol fuchsin [27]. 

In the negative stain, all samples must be allowed to dry before being inspected, which might take a substantial amount of time if a significant number of samples should be processed [21,25,26,27]. Because the most applied staining method (mZN) has disadvantages [22,23,24] and other diagnostic procedures might be costly; a reliable, accessible, rapid, and cost-effective technique for diagnosing *Cryptosporidium* should be considered. Therefore, the current study aims to improve the detection rate of *Cryptosporidium* oocysts in faecal samples via (a) testing different negative stains as a direct wet mount examination of oocysts with qualitative assessment and (b) assessing sensitivity and qualitative performance of toluidine blue dye and mZN in the detection of *Cryptosporidium* oocysts in faecal samples.

## 2. Materials and Methods

**Figure 1:**
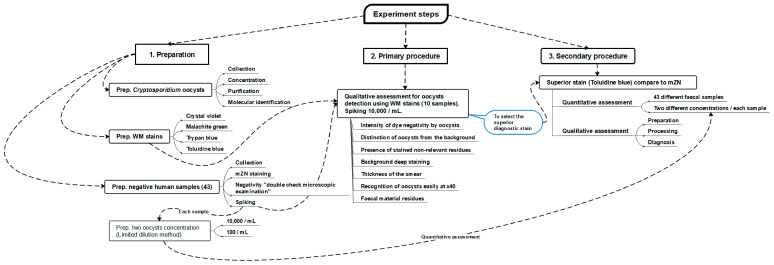
A flowchart illustrating the experiment’s stages. WM: Wet mount; mZN: Modified Ziehl Neelsen; Prep.: Preparation.

Figure 1 is a flowchart illustrating the experimental steps and phases of the current study.

### 2.1. The Oocysts Source

Neonatal calves with watery diarrhoea were sampled from a farm in El-Salhya El Gadeda, Ismailia, Egypt, where there is a documented history of recent cryptosporidiosis. [28]. Fifteen faecal samples were collected and transported immediately to the Laboratory of Medical Parasitology at the Suez Canal University, Ismailia, Egypt. A direct smear from each sample was spread on a slide and left to dry at room temperature. Using the mZN [29], stained faecal samples were examined microscopically for *Cryptosporidium* oocysts. Based on the average number of oocysts in 10 randomly selected high-power fields (HPFs), the intensity of excretion was evaluated semi-quantitatively according to Holzhausen et al. (2019) [30]: 0 = negative; 1 = 0.1–1 oocysts per field; 2 = 1.1–10 oocysts per field; 3 = 10.1–20 oocysts per field; 4 = 20.1–30 oocysts per field; 5 = more than 30 oocysts. 

For further molecular preparation, about 1 mL of the watery faecal specimens was transferred directly into 1.5 mL Eppendorf tubes and kept for additional molecular identification and confirmation at −20 °C.

### 2.2. Molecular Identification of Cryptosporidium Oocysts

The frozen faecal specimens were thawed in cold phosphate-buffered saline (PBS) and prepared for DNA extraction [28]. According to manufacturer instructions, about 200 µL of the obtained sediment was used for the DNA extraction using a Qiagen DNA stool mini kit (50, product of Germany) with minor modifications [28]. DNA aliquot was stored at −20 °C until PCR amplification. 

According to Bialek et al. (2002), the 18S rDNA gene of *C. parvum* was amplified using nested PCR [31] with minimal modifications [28]. The amplified products were visualized using a UV transilluminator after electrophoresis [31]. 

### 2.3. Concentration and Purification of Cryptosporidium Oocysts

Three faecal samples (score 4 to 5 = 20 − >30 oocysts/HPF) were preserved in 2.5% potassium dichromate (1:4, *v*/*v*) for further oocysts processing at 4 °C. 

As described [32,33,34], the faeces–potassium dichromate suspension was concentrated and purified with minor concentration modification (formalin–ethyl acetate procedure, in which formalin was replaced with 2.5% potassium dichromate). The purified oocysts suspension was collected in 15 mL tubes and stored at 4 °C in 2.5% potassium dichromate 1:4 *v*/*v*.

### 2.4. Counting of Purified Cryptosporidium Oocysts

Neubauer haemocytometer was used to determine the concentration counts of cryptosporidial oocysts in the previously prepared suspension, as described [35]. Oocysts with a final concentration of (1 × 10^6^ /mL) were incubated in sterile PBS (pH 7.4) with antibiotic suspension at 4 °C [28]. 

### 2.5. Stains Preparation for Qualitative Examination of Cryptosporidium sp. Oocysts

In the present experiment, wet mount staining techniques were utilized to stain the background bacteria and fungi in faeces but not *C. parvum* oocysts. Four wet mount stains were evaluated qualitatively for their ability to detect *Cryptosporidium* oocysts in the spiked faecal sample (negative faecal sample spiked with a known number of oocysts). Crystal violet (CV), malachite green (MG), toluidine blue (TolB), and trypan blue (TB) were the wet mount stains tested. Ten faecal samples loaded with 1 × 10^4^ *C. parvum* oocysts per millilitre were used to test the qualitative diagnostic ability of each stain. Seven criteria were used to evaluate each stain’s diagnostic capabilities for identifying oocyst-spiked samples. Two independent raters rated each stain based on its favourable or unfavourable qualities. After qualitative scoring evaluation, the superior diagnostic stain was selected for comparison with mZN stain in *C. parvum* oocysts detection. 

The CV staining preparation was as https://www.biognost.com/wp-content/uploads/2020/02/Crystal-Violet-powder-dye-IFU-V1-EN1.pdf instructions, accessed on 12 January 2022. The stock stain was then diluted with H_2_O 1:4 *v*/*v* because microscopic examination revealed a background black hue (preliminary study). The MG 3% was prepared following the CDC (2016) protocol [36]. TolB (C.I. 52040) was prepared in accordance with https://www.biognost.com/wp-content/uploads/2020/02/Toluidine-Blue-O-powder-dye-IFU-V5-EN1.pdf, accessed on 17 January 2022 According to https://cdn.gbiosciences.com/pdfs/protocol/Trypan_Blue_Solution.pdf, accessed on 17 January 2022, TB solution 0.4% was formulated, with the concentration increased to 0.8% to provide an appropriate colour with the surface area of the cover slide under microscopic examination (preliminary study). The mZN stain was prepared according to the suggested technique [37].

### 2.6. Sample Size Justification

Sample size calculations were performed using the equation described by Buderer (1996) [38]. Numerous studies have been conducted on the malachite green stain to detect *Cryptosporidium* oocysts; thus, it was used as a point of reference to determine the sample size. For naturally infected faecal samples, the sample size is calculated as 24 samples given that the expected sensitivity for detection of *Cryptosporidium* by the malachite green staining method is 99%, and the estimated prevalence of *Cryptosporidium* in Egypt was 15% [39], with a 10% margin of error and 95% level of confidence. Likewise, 43 samples will be large enough to yield a minimum sensitivity of 94% using the malachite green staining method for the detection of *Cryptosporidium* in the experimentally infected faecal samples, given that the prevalence is set at 50%, with a 10% margin of error and 95% level of confidence [40].

### 2.7. Negative Faecal Samples Preparation for Oocysts Spiking and Microscopic Examination

Forty-three negative faecal samples (Table 1), previously sieved, were concentrated (formalin–ethyl acetate technique) and initially analysed with mZN stain to ensure the negativity of *Cryptosporidium* oocysts and other coccidian protozoans, *Cyclospora cayetanensis*, and *Cystoisospora belli*. The direct wet mount detection results of *Cryptosporidium* oocysts were obtained using a double reading by two raters. The negative samples were used for a spiking experiment with known concentrations of *Cryptosporidium* oocysts and tested with the previously prepared stains (Section 2.5). The faecal samples were of human origin. We used a standard volume of 5 mL of formalin-fixed faecal sediments for all faecal samples.

**Figure 2:**
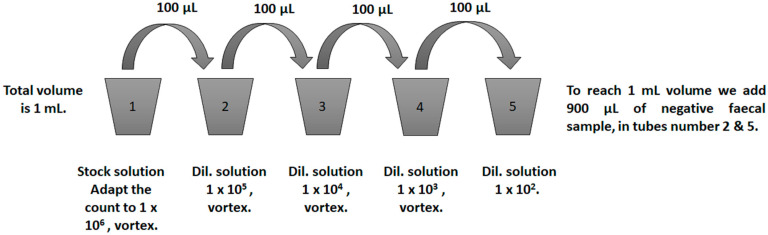
The limiting dilution method (LDM) was used to prepare spiked faecal samples with 1 × 10^4^ and 1 × 10^2^. Dil.: Diluted. Tubes 1, 3, and 4 were filled with a 1 mL suspension of *Cryptosporidium* oocysts in phosphate-buffered saline. Tubes 2, and 5 were filled with 100 µL suspension of *Cryptosporidium* oocysts in phosphate-buffered saline and 900 µL concentrated negative faecal samples.

Using the limiting dilution method (LDM) [41] tested for biological experiments, two different oocyst concentrations were prepared for each faecal sample. In brief, a final volume of 1 mL with 1 × 10^6^-PBS *C. parvum* oocysts was reached. The sample was vortexed, 100 µL was transferred to a new tube, and 900 µL of the negative faecal sample was added. The newly formed tube was then 1 mL of 1 × 10^5^. The procedure was continued until the desired two distinct concentrations for each sample were reached. Using LDM, concentrations of 1 × 10^4^ and 1 × 10^2^ were produced (Figure 2). The standardized final volume of the spiked sample was 1 mL. Because each faecal sample was spiked at two different concentrations, the total number of faecal samples for testing the stains was 86. TolB, which received the highest qualitative evaluation score among the previously tested stains (Section 2.5), will be contrasted with mZN in the following sections to identify *Cryptosporidium* oocysts.

### 2.8. Preparation of Slides for Staining Techniques from Spiked Samples

Before using the stain, the samples were centrifuged at 800 g for 5 min, and the supernatant was removed [40]. For TolB, a wet mount examination was performed on spiked samples. One drop (about 20 µL) of sediment and stain was combined and covered with a coverslip on the slide. Under ×400 and ×1000 magnification, *Cryptosporidium* oocysts were examined on microscopic slides. For the mZN stain, one droplet of sediment was applied to a slide and subsequently stained permanently. 

### 2.9. Quantitative and Qualitative Assessment of TolB and mZN

Using each stain (TolB and mZN), each sample was reported as negative or positive for *Cryptosporidium* oocysts in each concentration [40]. Two independent raters separately evaluated faecal smears; hence, two slides were prepared for each sample in each stain. If there was disagreement regarding the quantitative classification of the tested procedure, a third observer also examined the slides to reach a final decision by consensus [42]. 

A qualitative assessment was designed for each stain (TolB and mZN) regarding its preparation, processing, and diagnosis to be answered by the two independent raters.

### 2.10. Statistical Analysis

Descriptive statistics are summarized as mean ± standard deviation (SD) or percentages. As all samples were positive, no specificity analysis was conducted for the current study.

Inter-rater and inter-assay agreements were measured. Even though Cohen’s Kappa is the most used method, it has several limitations in the case of marginal values (i.e., high agreement but low-Kappa paradox) [43]. Gwet’s agreement coefficient (AC) has been suggested as a more robust agreement measure, representing the data more accurately [44]. Thus, we estimated Gwet’s AC for categorical variables and the two-way random-effects model intraclass correlation coefficient (ICC) for continuous outcomes. Coefficients were interpreted according to Landis and Koch (1977) [45].

To classify the four wet mount stains (TolB, MG, TB, and CV) for the detection of *Cryptosporidium* oocysts, ten samples were assessed by two raters according to seven parameters. Raters’ assessment was converted into scores (i.e., one if desirable and zero if undesirable), and then scores for each sample, each wet mount stain, and each rater were summed. For each stain, the score could range between 0 and 140, with higher values indicating a better qualitative assessment of the stain. Proportions of undesirable ratings were calculated, and results were interpreted as superior (<25% undesirable items), neutral (between 25 and 50% undesirable items), or inferior (>50% undesirable items) diagnostic methods, as shown in Table 2.

For the qualitative assessment evaluation of TolB and mZN, 21 questions were chosen based on items already evaluated for stains by previous researchers [20,23,26,37,40,46] and the authors’ prior knowledge and experience with staining techniques of *Cryptosporidium* oocysts [15,28]. The assessment criteria covered feasibility aspects during three phases: preparation, processing, and diagnosis. Six criteria were assessed for the preparation phase, twelve for the processing phase, and three for diagnosis.

For each item, two independent raters provided answers on a 5-point Likert scale ranging from 1 (i.e., strongly disagree) to 5 (i.e., strongly agree).

For unfavourable items (e.g., time-consuming preparation), scores were reverted so that a high score would reflect a favourable characteristic. Then, the average score between raters was calculated for each item, and total average scores were calculated for each phase and the overall evaluation. Scores could range between 6 and 30 for preparation, 12 and 60 for processing, and 3 and 15 for diagnosis. Hence, the overall score could range between 21 and 105, with higher values indicating better performance of the staining method.

The mean score difference between mZN and TolB was calculated. In addition, the mean value score for the total components was converted into percentages, and relative change was calculated. A good stain technique was assigned if there was a difference of 1.5 or more, at individual item analysis, and a relative change > 25% in the overall components. All statistical analysis was performed with Stata v16.

## 3. Results

### 3.1. Cryptosporidium Oocysts Purification Load and Molecular Characterization

**Figure 3:**
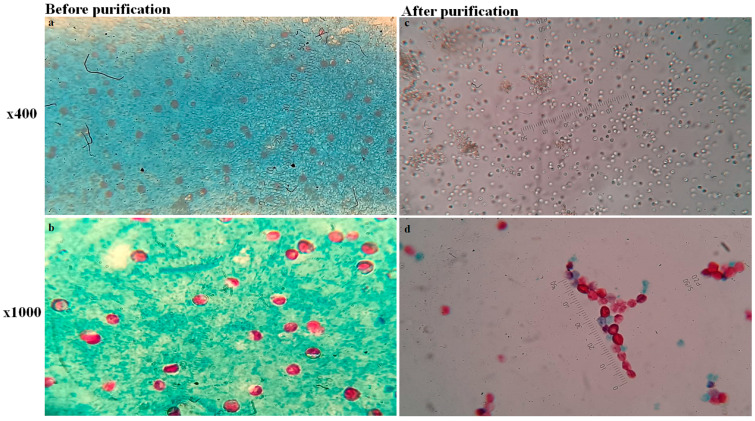
Oocysts of *Cryptosporidium* sp. before and after purification. (**a**,**b**) Before purification, a direct mZN–stained smear of faecal samples was photographed with ×400 dry and ×1000 oil lenses. After purification, (**c**) the wet mount examination of purified samples was photographed at ×400 and (**d**) mZN staining at ×1000. Each photograph had an ocular micrometre installed to lessen the variation in zooming.

In the current investigation, three faecal samples from a direct mZN-stained smear with a score of 4–5 were selected for purification. The samples contained a mean of twenty to more than thirty *Cryptosporidium* sp. oocysts per 1000× oil field. The gradient sucrose purification successfully collected many oocysts in the pure pellets (Figure 3).

The nested PCR technique was used and successfully amplified *Cryptosporidium* isolates. The three isolates all belonged to the species *C. parvum*. Electrophoresis was accomplished with a 285 bp product.

### 3.2. Qualitative Assessment of Wet Mount Stains in the Detection of Cryptosporidium Oocysts

The four wet mount stains (TolB, MG, TB, and CV) detected *Cryptosporidium* oocysts with varying qualitative results. TolB was the superior diagnostic stain for detecting *Cryptosporidium* oocysts in various faecal samples, yielding the lowest percentage of undesirable ratings (4%) (Table 3). TolB was able to differentiate oocysts from adjacent areas in faecal smears by dying other faecal elements, such as yeast cells, pollen grains, and digested food residues. TolB did not result in the precipitation of dye particles. It strongly stained the background, making oocysts detection simple. TolB facilitated the identification of the oocysts at ×40 power and in dense smears. TolB yielded identical results for diverse faecal materials.

**Figure 4:**
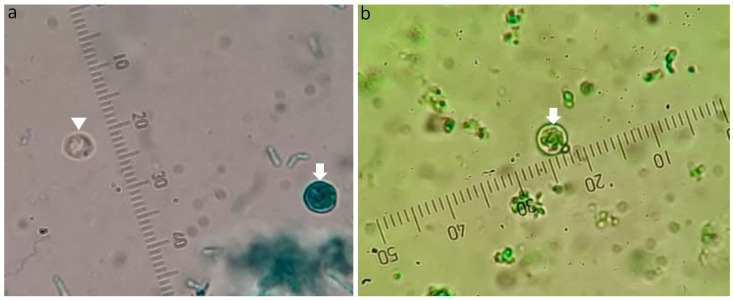
MG-stained faecal samples. Similar in size to oocysts, yeast cells reacted differently to the dye. (**a**) The photo combined yeast cell (arrow) that absorbed the malachite green colour, and *Cryptosporidium* oocyst negatively stained (arrowhead). (**b**) The photo contained yeast cell only that exhibited an adverse reaction and appeared refractile. A 1000× microscopic lens captured the photos. An ocular micrometre was fitted in each photo to reduce the difference in zooming.

According to TolB’s qualitative score, all items were desirable. MG had a regular oocysts’ diagnostic score, with 45% of undesirable ratings (Figure 4). TB and CV scored as inferior stains for diagnosing *Cryptosporidium* oocysts, with a maximum score of undesirable ratings of 73% and 100%, respectively.

Notably, the intraclass correlation coefficient showed almost perfect agreement between rater’s scores (Table 4).

**Figure 5:**
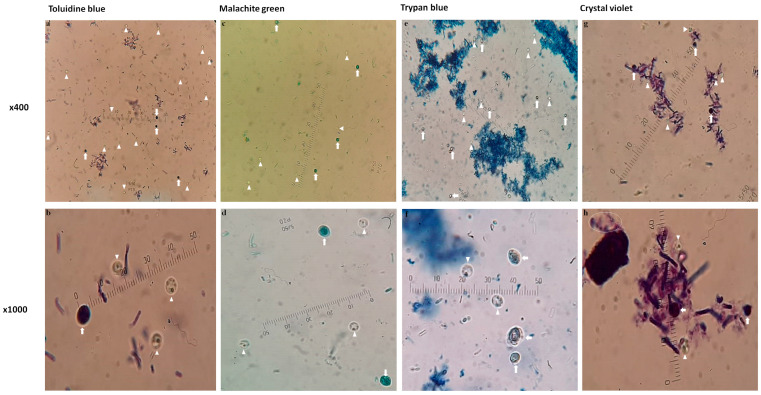
Evaluation of the qualitative diagnostic abilities of various wet mount stains in detecting *Cryptosporidium* oocysts. Images were taken at two different magnifications of a faecal sample containing *Cryptosporidium* oocysts and spores that resemble fungi (×40 and ×1000). Oocysts were identified using four different stains. Toluidine blue (**a**,**b**), malachite green (**c**,**d**), trypan blue (**e**,**f**) and crystal violet (**g**,**h**) were applied as a wet mount technique. The arrowhead (triangle) refers to the shape of *Cryptosporidium* oocysts in each stain. The arrow identifies the fungal spore in each stain. The elliptical shape determines the stain precipitation (**h**). An ocular micrometre was fitted in each photo to reduce the difference in zooming.

The qualitative diagnostic capabilities of the four wet mount stains for detecting *Cryptosporidium* oocysts are presented in Figure 5. To demonstrate the microscopic quality of *Cryptosporidium* oocysts detection in all stains, a single faecal sample containing fungal spores similar in size and shape to *Cryptosporidium* oocysts was selected.

### 3.3. Quantitative Assessment of TolB and mZN

**Figure 6:**
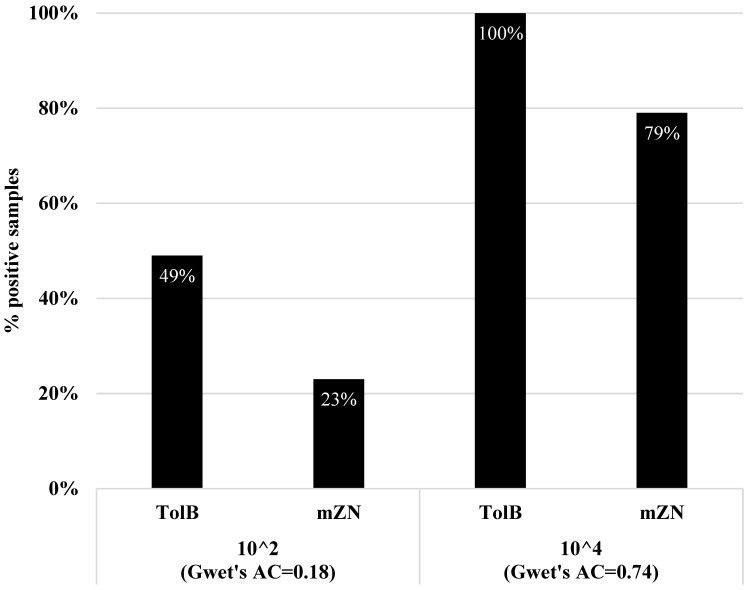
The proportion of positive samples identified by TolB and mZN staining.

A set of 43 faecal samples with two different oocysts concentrations was established. After consensus, TolB recognized all samples as positive for *Cryptosporidium* at a concentration of 10^4^ (sensitivity = 100%), while mZN evaluated 34/43 samples as positive (sensitivity = 79%). At a concentration of 10^2^, TolB detected twice as many positive samples as mZN did (sensitivity = 49% and 23%, respectively) (Figure 6). 

Inter-rater and inter-assay percentages of agreement and Gwet’s AC are reported in Table 5. Inter-rater agreement was substantial for samples analysed with mZN stain at concentrations 10^2^ and 10^4^ (Table 5, a and b) and almost perfect for samples analysed with TolB stain at concentrations 10^4^ (Table 5, c). In contrast, agreement was poor at concentration 10^2^ (Table 5, d).

For both stain methods and raters, inter-assay agreement (by concentration) was poor to slight (Table 5, e–h). Finally, the inter-assay (by staining way) agreement was fair to slight at concentration 10^2^ for raters, respectively (Table 5, i and j), while substantial at concentration 10^4^ in both raters (Table 5, k and l).

### 3.4. Qualitative Assessment of TolB and mZN

The qualitative assessment of TolB and mZN concerning preparation, processing, and diagnostic criteria are presented in Table 6. Overall, TolB was more favourable than mZN in all three phases evaluated, improving the performance by 43% (varying from a 27% better rating for the preparation phase to a 57% better rating for the diagnosis components). However, mZN showed good performance for permanent staining and for not being affected by the freshness of the faecal sample. Furthermore, both methods showed similar ratings regarding cost-effectiveness. 

**Figure 7:**
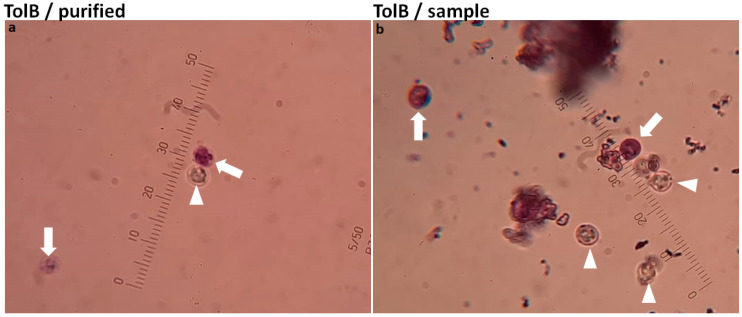
Ability of TolB to differentiate between viable and non-viable *Cryptosporidium* oocysts. Arrowhead refers to viable oocysts, and arrow refers to non-viable oocysts. A 1000× microscopic lens captured the photos. Purified oocysts were used in (**a**) and a spiked faecal sample were used in (**b**). An ocular micrometre was fitted in each photo to reduce the difference in zooming.

After two months of spiking the samples, TolB could no longer detect the same quantity of oocysts in the same samples, indicating that the freshness of the sample had an impact on its diagnostic ability. Several oocysts seemed to absorb the stain and took on the appearance of the background. The fact that TolB can be employed as a viability stain in testing for other drug treatment experiments stains, however, emerged as a benefit from this (Figure 7).

**Figure 8:**
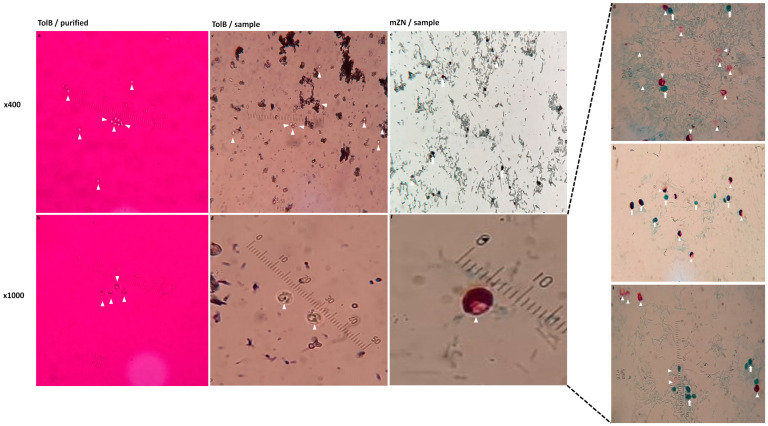
The demonstration difference between wet mount TolB and permanent mZN in detecting *Cryptosporidium* oocysts is illustrated. Images were taken at two magnifications (×400 and ×1000) from a purified sample and one of the spiked samples. The arrowhead (triangle) points to the *Cryptosporidium* oocysts, and the arrow indicates the fungal spores in the sample. TolB: Toluidine blue (**a**–**d**). mZN: modified Ziehl–Neelsen (**e**–**i**). Purified oocysts were used in (**a**,**b**), and a spiked faecal sample was used in (**c**–**i**). An ocular micrometre was fitted in each picture to reduce the difference in zooming.

TolB demonstrated that *Cryptosporidium* oocysts have a typical size (4–6 m) and content (four sporozoites, crescent shape). With their stain resistance, oocysts appeared with a sharp gloss against a stained burgundy background, and they were highlighted. However, similar-sized fungi swallowed the TolB stain and were concealed in the ground. At the microscopic dry magnification of ×400, it was simple and easy to detect TolB oocysts (Figure 8a,c). 

The mZN, on the other hand, exhibited varied staining of *Cryptosporidium* oocysts and fungal spores that could only be identified at a microscopic oil magnification of ×1000 (Figure 8g–i). The staining of mZN-stained oocysts ranged from empty hollow circles to a light-pink stain to a deep-rose stain, while the staining of fungal spores ranged from green to purple to dark red (Figure 8g–i). The various staining of oocysts and fungal spores confused raters regarding the positivity/negativity of samples, especially when the spores resembled the size of *Cryptosporidium* oocysts and took the variable atypical staining colour. It became challenging to reach a final decision due to several uncertainties; therefore, a third expert consultation was considered (Table 6; Figure 8g–i). At the dry power ×400, it was challenging to differentiate between oocysts and fungi (Figure 8e).

## 4. Discussion

Diagnosing *Cryptosporidium* oocysts using microscopy is challenging among investigators (technicians in diagnostic laboratories and researchers in research laboratories) [1,24,47]. mZN, immunochromatography (ICT), immunofluorescence, and PCR are methods applied by diagnostic laboratories in developed countries to identify *Cryptosporidium* sp. In specific laboratories, mZN is only used to confirm the accuracy of the ICT results [24,48,49]. Cryptosporidiosis epidemics continue to be misdiagnosed and underestimated despite the presence of facilities in industrialized nations [6,7,8,9,50]. In contrast, developing countries frequently look for low-cost diagnostic methods to identify *Cryptosporidium* sp. using staining techniques like mZN [19,20]. The only predictor of the final patient diagnosis of cryptosporidiosis is, thus, anticipated to be the investigator’s experience. In other countries, however, *Cryptosporidium* is not even on the list of parasites that should be examined, so it goes undetected [1,18]; Karanis, pers. com, accessed on 13 March 2022.

To effectively diagnose cryptosporidial infections, diagnostic techniques must be quick, affordable, accurate, and sensitive. The one-drop faecal sediment and one-drop stain method for preparing specimens is easy, safe, and practical [51]. The wet mount technique, hence, will be a straightforward method that any investigator can execute.

The diagnostic performance of four wet mount stains to identify *Cryptosporidium* oocysts in faecal samples was assessed in the current experiments. For identifying *Cryptosporidium* oocysts in various spiked faecal samples, TolB outperformed MG, TB, and CV. TolB, as far as the authors are aware, was first employed to identify *Cryptosporidium* oocysts. Oocysts in spiked faecal samples were unstained, while all sediments (yeasts/moulds, vegetables, bacteria, etc.) absorbed the stain colour. With different faecal sample materials, TolB consistently reacted positively. However, MG, TB, and CV were classified with undesirable characteristics, even though these stains had previously been utilized in other studies with promising outcomes [26,27,40,52,53]. The source of the faeces—human or animal—and, occasionally, which animal seem to impact how well the preceding stains turn out [40]. The current study’s human faecal samples had a lot of yeasts, which made diagnosis challenging, especially when they were comparable in size to oocysts and could cause a false-positive result for *Cryptosporidium* infection. Even though MG was scored as a regular stain for detecting *Cryptosporidium* oocysts, specific yeasts reacted as negatively stained objects, causing the investigators to misdiagnose the oocysts (Figure 4). It has also been observed that using MG to detect *Cryptosporidium* in birds can result in false-negative results [52]. 

In the current investigations, TB was rated as an inferior stain, and yeasts did not usually pick up the stain (Figure 5e,f), making diagnosis difficult. The background debris failed to absorb the stain adequately, and, therefore, it appeared lighter, and the oocysts did not stand out as prominently as those with TolB. According to another investigation, TB was an effective stain for wet mount detection of *Cryptosporidium* oocysts in purified samples [28]. The detection of oocysts by TB in faecal debris samples appears distinct from that in purified, debris-free samples.

CV stain was the inferiorest stain based on the qualitative evaluation in the current examination. The CV stain was a dense pigment that precipitated dye particles, which complicated the diagnosis by decreasing the refractivity of the oocysts’ structures (Figure 5g,h). The preparation of a dense smear makes the identification of oocysts particularly challenging. However, CV has been reported to enhance the oocysts’ refractivity, despite being the least effective stain for diagnosing a positive infection [27].

In the present study, two distinct concentrations (10^2^ and 10^4^) of TolB and mZN were used to detect *Cryptosporidium* oocysts. TolB shows a more significant proportion of positive infections at both concentrations than mZN. TolB was a sensitive stain that detected oocysts in all spiked samples at 10^4^. However, roughly 20% of mZN samples were misdiagnosed. TolB detected positivity in almost half of the samples infected at concentration 10^2^ compared to one-third by mZN. Even though mZN is widely reported as the ‘gold standard’ for detecting oocysts [19,20,21], the stain’s reliability remains a concern.

Both investigators reported difficulty in detecting oocysts with both stains at lower concentration (10^2^) with poor agreement, particularly with mZN. Spiked samples with 10^2^ were regarded as low when only one drop per slide was analysed [40]. It is typically challenging to distinguish nonviable *Cryptosporidium* oocysts from yeasts of the same size in infected human faeces using wet mount stains. However, if a purified sample is used, the examiner can easily differentiate between viable and nonviable oocysts. Yeast typically does not exist in freshly purified samples under these conditions. 

In contrast, it would be challenging for the untrained or inexperienced person to distinguish between oocysts and similar yeasts in infected human faeces (Figure 5e,f). The morphology of oocysts is highly distinguishable from yeasts of comparable size to the trained eye (Figure 5f, arrows and arrowheads). Therefore, when staining, the experiences of the investigators should consider important diagnostic factors, like oocyst and yeast morphology, their stain response, and different sample debris variation. 

The disagreement between raters in some quantitative points prompted additional qualitative scoring of each stain’s weakness limb. Preparation, processing, and diagnosis were evaluated for both stains. TolB outperformed mZN in almost every item assessed (Table 6). The TolB technique is more straightforward and less expensive than mZN staining because it requires only one stain. This staining takes less than half as long as the mZN staining, and the sensitivity can be increased by examining specimens under ×400 magnification.

TolB–*Cryptosporidium* oocysts (Figure 8a–d) appeared as unstained, strongly refractive, round to oval structures of about 3 to 6 µm against a burgundy background. Internal structures were slightly visible as darker specks inside the oocysts. Oocysts were nearly identical in form, size, and overall appearance. The burgundy colour was very efficiently absorbed by yeast and faecal debris. 

mZN–*Cryptosporidium*-stained oocysts (Figure 8e–i) had a high degree and proportion of staining that varied with each oocyst. Oocysts internal structures absorbed the stain to differing degrees; some may contain crescent-shaped sporozoites, while others may appear ragged. Holes or spaces in the counterstain indicated the presence of “ghost” oocysts, which are oocysts that have not been stained. Additionally, yeasts and faecal debris were stained in various colours (red, purple, and green), and some of the yeasts were in the size range of oocysts. Similar reports mentioned these observations about the mZN that required intensive training and experience to interpret the results [21,40,46].

Nevertheless, as with any stain, there are some concerns. Regarding TolB–oocysts examination, certain aspects must be carefully considered: (i) the prepared slide is a wet mount slide that will dry out in 10 to 15 min; (ii) the faecal sample must be fresh since oocysts that have been stored for a long time lose viability and start to absorb the stain, making them impossible to distinguish from yeasts and faecal debris; (iii) the dye colour reacts with the potassium dichromate colour (faecal sample preservative) and obscures the detection of oocysts; therefore, it is advised that one should apply multiple washes of PBS/saline/formalin/water to potassium dichromate-preserved samples before using TolB; (iv) thickness of the smear adversely affects the visibility of the oocysts; (v) training of the investigator is required before using TolB stain on a positive control; (vi) utilization of ×400 dry lens provides a quick, simple, and straightforward view of oocysts detection; (vii) using mZN staining, doubtful or equivocally positive samples can be confirmed.

mZN is effective for diagnosing positive instances when three factors are present. (i) high–quality staining ingredients. (ii) an expert in diagnosing *Cryptosporidium* oocysts. (iii) enough oocysts in the investigated faecal sample. The examination of oocysts using the mZN method is hampered by crucial obstacles: (i) application defeasibility to a large number of samples per day in diagnostic laboratories, mainly when there is a technician shortage [1]; (ii) requirement of modification of the de-staining process by sulfuric acid (H_2_SO_4_), which differs by sample debris difference and thickness of the smear [54]; (iii) several stool samples over several days are required [21]. 

Other species, such as *Cryptosporidium hominis*, a common species found in humans, were inapplicable to the current investigation. Due to the high cost of PCR in an unfunded project, the PCR could not be conducted to ensure the negativity of samples; instead, the microscopic examination was utilized.

Several recommendations will aid investigators using the mZN staining technique: (i) interpret *Cryptosporidium* positivity when more than five conventional oocysts are observed on an examined slide. (ii) add oil to the slide when examining with an ×400 dry lens to see oocysts more distinctly. However, it requires a trained eye. (iii) repeat the staining if red yeasts and bacteria appear in the background counterstain. (iv) direct faeces smear stains better than sedimented faeces smear. (v) animal faecal samples stain differently than human faecal samples. (vi) if you suspect the infection in a clinically positive patient, repeat stool sample examinations on consecutive days. (vii) include a positive control slide while you are staining your sample set. (viii) consult a senior expert when you have doubts about the results, or send the permanent slide to a reference lab for verification.

## 5. Conclusions

TolB is a more practical, safe, and sensitive procedure than mZN and other wet mount stains for detecting *Cryptosporidium* oocysts in human stool samples. Less-experienced microscopists can accurately diagnose cryptosporidial infections with TolB because oocysts can be distinguished from yeasts with relative simplicity. Due to the wide range of results that parasitology/infectious disease laboratories can produce when diagnosing *Cryptosporidium*, the TolB wet mount staining procedure should significantly improve microscopic diagnoses and cryptosporidial infection detection. The preliminary findings of this investigation permit us to proceed with the interlaboratory validation of the TolB.

## Figures and Tables

**Table 1:** Negative faecal samples’ data.

ID	Gender	Age in Years	Consistency	ID	Gender	Age in Years	Consistency
1	Female	4	Formed	23	Female	34	Diarrhoea
2	Male	7	Diarrhoea	24	Female	57	Formed
3	Male	3.5	Formed	25	Female	56	Diarrhoea
4	Female	8	Formed	26	Female	51	Formed
5	Female	6	Diarrhoea	27	Female	40	Formed
6	Female	35	Formed	28	Female	34	Formed
7	Male	38	Formed	29	Male	37	Formed
8	Male	9	Formed	30	Male	62	Formed
9	Female	7	Formed	31	Female	41	Formed
10	Male	40	Formed	32	Female	29	Formed
11	Male	4	Diarrhoea	33	Female	13	Formed
12	Female	8	Formed	34	Female	26	Formed
13	Female	4.5	Formed	35	Female	45	Formed
14	Male	3	Formed	36	Male	9	Diarrhoea
15	Male	10	Formed	37	Male	4	Formed
16	Male	3	Formed	38	Female	43	Formed
17	Female	12	Formed	39	Female	36	Formed
18	Female	42	Formed	40	Male	6	Diarrhoea
19	Female	22	Formed	41	Female	8	Formed
20	Female	37	Formed	42	Male	48	Formed
21	Female	28	Diarrhoea	43	Male	2.5	Formed
22	Female	44	Formed				

**Table 2:** Scoring system for the qualitative evaluation of various wet mount stains for the detection of *Cryptosporidium* oocysts in spiked faecal samples.

Qualitative Variables	Qualitative Assessment		Explanation of Undesirable Characteristics
	Desirable	Undesirable	
Intensity of dye negativity by oocysts	1	0	Cannot discriminate the oocysts from surrounding areas in faecal smears
Distinction of oocysts from the background	1	0	Dying other faecal elements such as yeast cells, pollen grains, or digested food residues
Presence of stained non-relevant residues	1	0	Produce precipitation of dye particles, which is a main concern for *Cryptosporidium* diagnosis, especially when sizes and shapes of residues are similar to the oocysts.
Background deep staining	1	0	Background stains light and oocysts detection become difficult
Recognition of oocysts easily at ×40	1	0	Cannot recognize the oocysts at power ×40.
Thickness of the smear	1	0	Cannot recognize the oocysts in thick smear
Faecal material residues	1	0	Produces distinct outcomes for various faecal materials.

Interpretation: >50% of faecal smears with any “undesirable characteristics” = inferior diagnostic method; 25–50% of faecal smears with any “undesirable” characteristics = regular diagnostic method; <25% of faecal smears with any “undesirable” characteristics = superior diagnostic method.

**Table 3:** Qualitative scoring of various wet mount stains for detecting *Cryptosporidium* oocysts in spiked faecal samples.

Score	TolB	MG	TB	CV
Mean (±SD)	6.8 (±0.6)	3.9 (±3.6)	1.9 (±2.8)	0
Median (IQR)	7 (7–7)	7 (0–7)	0 (0–5)	0
Total (range 0–140)	135	77	38	0
% Undesirable	4	45	73	100
Interpretation	Superior	Regular	Inferior	Inferior

TolB: toluidine blue; MG: malachite green; TB: trypan blue; CV: crystal violet; SD: standard deviation; IQR: interquartile range.

**Table 4:** Intra-class correlation coefficients between raters for the four wet mount stains.

Wet Mount Stain	Individual	Average
TolB	0.84 *	0.91 *
MG	0.82 *	0.90 *
TB	0.94 *	0.97 *
CV	NA	NA

TolB: toluidine blue; MG: malachite green; CV: crystal violet; TB: trypan blue; NA: not applicable. * statistically significant at *p* < 0.05.

**Table 5:** Inter-rater and inter-assay percentage agreement and Gwet’s agreement coefficient.

Test Comparison	Sensitivity %	Agreement %	Gwet’s Agreement Coefficient	Interpretation
(a) TolB × 10^2^ rater 1 vs. rater 2	39.5 vs. 48.8	48.8	−0.01	Poor agreement
(b) TolB × 10^4^ rater 1 vs. rater 2	93.0 vs. 100	93.0	0.93 *	Almost perfect
(c) mZN × 10^2^ rater 1 vs. rater 2	23.3 vs. 23.3	81.4	0.71 *	Substantial agreement
(d) mZN × 10^4^ rater 1 vs. rater 2	81.4 vs. 79.1	93.0	0.90 *	Substantial agreement
(e) Rater 1 TolB × 10^2^ vs. ×10^4^	39.5 vs. 93.0	41.9	−0.05	Poor agreement
(f) Rater 2 TolB × 10^2^ vs. ×10^4^	48.8 vs. 100	48.8	0.17	Slight agreement
(g) Rater 1 mZN × 10^2^ vs. ×10^4^	23.3 vs. 81.4	37.2	−0.25	Poor agreement
(h) Rater 2 mZN × 10^2^ vs. ×10^4^	23.3 vs. 79.1	44.2	−0.12	Poor agreement
(i) Rater 1 × 10^2^ TolB vs. mZN	39.5 vs. 23.3	60.5	0.31	Fair agreement
(j) Rater 2 × 10^2^ TolB vs. mZN	48.8 vs. 23.3	55.8	0.18	Slight agreement
(k) Rater 1 × 10^4^ TolB vs. mZN	93.0 vs. 81.4	79.1	0.73 *	Substantial agreement
(l) Rater 2 × 10^4^ TolB vs. mZN	100 vs. 79.1	79.1	0.74 *	Substantial agreement

mZN: modified Ziehl–Neelsen; TolB: toluidine blue, * Statistically significant at *p* < 0.05.

**Table 6:** Qualitative scoring of TolB versus mZN.

Items	Variables	Stain		TolB-mZN (Relative Change%)	Favorable Stain
		TolB (%)	mZN (%)		
Preparation	Safe	5	3.5	1.5	TolB
	Practical	5	3.5	1.5	TolB
	Time-consuming *	4.5	1.5	3	TolB
	Permanent stain	1.5	5	−3.5	mZN
	Cost-effective	3.5	3	0.5	Both
	Wet mount stain	5	1.5	3.5	TolB
	Total (range 6–30)	24.5 (82%)	18 (60%)	6.5 (27%)	TolB
Processing	Time-consuming *	4.5	1	3.5	TolB
	The stain has variable background coloration *	4.5	1	3.5	TolB
	The yeast takes up the stain	5	3.5	1.5	TolB
	The stain clearly differentiates the yeast from the oocysts	5	2.5	2.5	TolB
	The use of microscopic power ×40 is accessible to identify the oocysts	5	2	3	TolB
	It is easy to identify the oocysts among faecal sediment	5	3	2	TolB
	The oocysts preserve its features	5	3.5	1.5	TolB
	The sample sediment characteristics affect the staining *	4.5	1.5	3	TolB
	The stain gets affected by the freshness of the faecal sample *	2	3.5	−1.5	mZN
	The staining technique can be applied in the field	5	3	2	TolB
	The technique can be repeated on the same sample	5	3.5	1.5	TolB
	Using faecal concentration method affects the result of the staining technique *	4.5	1.5	3	TolB
	Total (range 12–60)	55 (92%)	29.5 (49%)	25.5 (47%)	TolB
Diagnosis	Diagnosis will vary with different investigators *	4.5	2	2.5	TolB
	Diagnosis have sometimes doubt regarding the typical oocysts shape *	4.5	1.5	3	TolB
	It is easy to interpret the slide via visual inspection	5	2.5	2.5	TolB
	Total (range 3–15)	14 (93%)	6 (40%)	8 (57%)	TolB
Total (range 21–105)		93.5 (89%)	53.5 (51%)	40 (43%)	TolB

* Original scores were reverted.

## Data Availability

The data presented in this study are available within the article.
